# Type 2 Diabetes Impairs Alveolar Socket Healing: Immunohistochemical Analysis of Del‐1, IL‐17, RANKL, and OPG Expression in a Rat Model

**DOI:** 10.1155/ijod/2332997

**Published:** 2026-05-30

**Authors:** Kübra Güler, Emine Pirim Görgün, Özhan Karataş, Serkan Bolat

**Affiliations:** ^1^ Department of Periodontology, Faculty of Dentistry, Uskudar University, Istanbul, Türkiye, uskudar.edu.tr; ^2^ Department of Periodontology, Faculty of Dentistry, Sivas Cumhuriyet University, Sivas, Türkiye, cumhuriyet.edu.tr; ^3^ Department of Pathology, Faculty of Veterinary Medicine, Sivas Cumhuriyet University, Sivas, Türkiye, cumhuriyet.edu.tr; ^4^ Department of Biochemistry, Adana City Hospital, Adana, Türkiye

**Keywords:** cytokines, immunohistochemistry, tooth extraction, type 2 diabetes mellitus, wound healing, X-ray micro-computed tomography

## Abstract

**Background:**

Type 2 diabetes (T2D) is known to impair bone healing, yet the molecular mechanisms underlying this process in alveolar sockets remain insufficiently understood. This study aimed to evaluate the expression of some key regulatory proteins related to angiogenesis and bone remodeling during alveolar socket healing in a diabetic rat model.

**Methods:**

Thirty‐three male Sprague‐Dawley rats were divided into control and T2D groups. T2D was induced by a high‐fat diet combined with low‐dose streptozotocin. Following mandibular molar extraction, animals were sacrificed at days 7, 14, and 28 postextraction. Alveolar bone tissues were examined using microcomputed tomography (micro‐CT) for structural analysis. Immunohistochemical staining was performed to assess the expression patterns of developmental endothelial locus‐1 (Del‐1), interleukin 17 (IL‐17), osteoprotegerin (OPG), and receptor activator of nuclear factor kappa‐B ligand (RANKL) with semiquantitative evaluation. Parametric tests were used for group comparisons and correlation analyses.

**Results:**

Micro‐CT analysis revealed reduced bone volume and trabecular integrity in the T2D group compared to controls. Del‐1 was localized to vessel walls within the bone marrow, IL‐17 was found in both bone tissue and marrow, while RANKL and OPG were detected in osteoblasts and osteocytes. Protein staining was mild or absent in diabetic groups (*p*  < 0.05) and declined over time.

**Conclusion:**

T2D negatively affects bone healing, potentially by disrupting the expression balance of key proteins involved in angiogenesis and bone remodeling. These findings highlight the need for further research on molecular targets in diabetic bone repair.

## 1. Introduction

Type 2 diabetes mellitus (T2D) is rapidly expanding worldwide, with global prevalence projected to increase from 5.9% to 9.5% between 2021 and 2050, potentially affecting over 1.27 billion people [1]. This chronic metabolic disorder is characterized by persistent hyperglycemia resulting from impairments in insulin secretion or insulin function or both. T2D is associated with both periodontal diseases and significant disruptions in bone metabolism, leading to impaired bone homeostasis and reduced bone quality, which exacerbates the risk of oral health complications, including tooth loss [2].

The loss of teeth due to periodontal disease or other complications in diabetic patients detrimentally impacts both functional and esthetic aspects of oral health, impairing mastication and affecting the quality of life [3]. Dental implants, a widely accepted intervention for edentulism, restore both esthetics and masticatory function. However, the success of dental implant procedures, as in osseointegration, is contingent upon both implant surface modifications and the quality of the alveolar bone, which studies show is compromised in individuals with T2D due to the impaired bone remodeling processes associated with the disease [4]. While the mechanisms regulating bone metabolism are not yet fully elucidated [5], their role in alveolar socket healing under diabetic conditions remains particularly underexplored, highlighting the need for further investigation. Understanding the interactions between diabetes‐related factors and bone regeneration will facilitate the development of more effective therapeutic strategies, ensuring optimal outcomes for diabetic patients undergoing dental implant treatment. Bone healing is influenced by various regulatory proteins involved in angiogenesis, inflammation, and bone remodeling, including developmental endothelial locus‐1 (Del‐1), interleukin‐17 (IL‐17), osteoprotegerin (OPG), and activator of the nuclear factor kappa beta ligand (RANKL) [6–8]. However, their expression in T2D alveolar socket healing remains understudied.

Del‐1, mostly studied for its anti‐inflammatory behaviors in dentistry [9], was initially recognized for its role in angiogenesis [8]. Given the importance of angiogenesis in socket healing and the vascular complications associated with T2D [10], a link between Del‐1 and diabetic socket healing is suggested. IL‐17 is a bone‐resorptive cytokine linked to diabetes [11]. OPG and RANKL are vital for bone metabolism [6, 7] and are expressed by osteoblasts and osteocytes during alveolar socket healing [5, 12]. OPG inhibits osteoclastogenesis and bone resorption by blocking RANKL [6]. IL‐17 upregulates RANKL expression [13], while Del‐1 was reported to inhibit it [8].

Given the high prevalence of T2D and a significant number of undiagnosed cases [14], diabetic tooth extractions are common. While modern medicine allows for good glycemic control, it doesn’t necessarily improve bone quality in T2D patients [15]. Therefore, new methods are needed to enhance healing in diabetic patients.

To the best of our knowledge, this is the first study to simultaneously evaluate Del‐1, IL‐17, OPG, and RANKL expression during alveolar socket healing under T2D conditions. While previous studies have examined these markers individually in periodontal inflammation or systemic bone diseases, their combined assessment in the specific context of diabetic extraction socket healing has not been reported.

As a preliminary investigation, this study seeks to establish foundational insights for future research. Using semiquantitative immunohistochemical analysis, it investigates the release of specific proteins (Del‐1, IL‐17, OPG, and RANKL) during T2D extraction socket healing to determine whether their expression differs from that in nondiabetic controls.

## 2. Materials and Methods

### 2.1. Ethical Approval and Establishment of the T2D Model

This manuscript adhered to the ARRIVE guidelines and received ethical approval from the Sivas Cumhuriyet University Animal Experiments Ethics Committee (Approval Number: 65202830‐050.04.04‐484).

**Figure 1:**
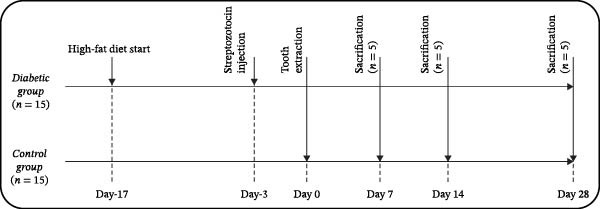
Study design.

The T2D model used in this study is documented in the literature [16]. It was established by combining a high‐fat diet with a low‐dose streptozotocin injection, which induces insulin resistance and mimics T2D metabolism [16]. Considering that alveolar socket healing is established on day 28 of tooth extraction in rats [12], we used the timepoints given in Figure 1 for our research.

### 2.2. Animal Count and Dietary Protocols

Sample size was calculated using G^∗^Power based on immunohistochemical scoring as the primary outcome [12], with five animals per subgroup (*α* = 0.05, power = 0.9, Cohen’s *d* > 0.8). The study design is summarized in Figure 1, and group details are given in Table 1.

**Table 1:** Description of the study groups.

Main groups	Subgroups	Description of the study groups (*n*)
Control	C7	Control group to be investigated at 7 days of alveolar socket healing (*n* = 5)
	C14	Control group to be investigated at 14 days of alveolar socket healing (*n* = 5)
	C28	Control group to be investigated at 28 days of alveolar socket healing (*n* = 5)
Diabetic	D7	Diabetic group to be investigated at 7 days of alveolar socket healing (*n* = 6)
	D14	Diabetic group to be investigated at 14 days of alveolar socket healing (*n* = 6)
	D28	Diabetic group to be investigated at 28 days of alveolar socket healing (*n* = 6)

A total of 33 healthy, with no history of procedures, male Sprague‐Dawley rats (16 weeks old, 310–360 g) were obtained from the Sivas Cumhuriyet University Experimental Animal Research and Application Center, and all procedures were conducted within the same facility. Rats were housed under controlled conditions (humidity, temperature, and 12‐h light/dark cycles).

After a 14‐day acclimatization period, rats were randomly divided into diabetic (*n* = 18) and control (*n* = 15) groups. The diabetic group had three extra rats due to higher vulnerability.

The diabetic model used in this study was developed by feeding rats a high‐fat diet in order to induce insulin resistance in combination with a low dose of intraperitoneal streptozotocin, resulting in partial cell dysfunction and lowered insulin secretion. This model is widely used [16] for investigating the type 2 diabetic state due to the model showing noninsulin dependency, insulin resistance, hyperglycemia, and abnormal lipid profiles.

Both groups had free access to water. The diabetic group had a high‐fat diet (Research Diets, Cat. Number: D12451), and the control group had a standard laboratory rodent diet (Optima Yem, Cat. Number: 45843) for 2 weeks.

Diabetes was then induced in the diabetic group via a single intraperitoneal injection of streptozotocin (30 mg/kg), dissolved in 0.1 M sodium citrate buffer (pH 4.5). The control group received the vehicle only.

All animals maintained their diets and had ad libitum access to food and water. Food and water intake were recorded daily.

### 2.3. Postinduction Glucose Testing, OGTT, and Animal Allocation

Following T2D induction, nonfasting blood glucose was measured via the tail vein using a glucometer (Accu‐Chek Active, Roche). Rats with glucose values ≥300 mg/dL were considered diabetic.

Within 72 h, an oral glucose tolerance test (OGTT) was conducted by administering 2 g/kg glucose via intragastric gavage. Blood samples were taken from the tail vein at 0, 60, and 120 min, and glucose was measured using the same glucometer. Results were evaluated per standard criteria [17].

After OGTT, animals were randomly divided into six subgroups, as shown in Table 1, with group names indicating control (C) or diabetic (D) status and days postextraction. Body weight, fluid intake, and energy consumption were regularly monitored in the longest surviving groups (C28 and D28) due to different termination time points.

### 2.4. Surgical Procedures and Termination of the Experiment

On day 0 (Figure 1), rats were anesthetized (xylazine 5 mg/kg and ketamine 90 mg/kg, i.p.). The right mandibular molar was luxated and extracted. Ground diets were given for 2 days postextraction to promote healing.

The experiment was terminated gradually at time points described in Figure 1. One animal from each diabetic group (D7, D14, and D28) was lost (two due to hyperglycemia, one during anesthesia). No animals were lost in the control groups. Final group counts were C7 (*n* = 5), C14 (*n* = 5), C28 (*n* = 5), D7 (*n* = 5), D14 (*n* = 5), and D28 (*n* = 5).

After overnight fasting, rats were sacrificed via overdose on scheduled days. Before sacrifice, 2 mL of blood was collected by cardiac puncture. Mandibles were harvested and fixed in 10% neutral‐buffered formalin.

### 2.5. ELISA and Biochemistry

Blood samples were allowed to coagulate at room temperature for 30 min and then centrifuged at 4000 rpm for 10 min. The serum was transferred to secondary tubes and stored at ‐80°C until analysis. The researcher conducting the assays was blinded to the sample identity.

Fasting insulin levels were measured using a quantitative ELISA kit (BT Lab, Catalog Number: E0707Ra) following the manufacturer’s instructions. Samples and reagents were equilibrated to room temperature, and all standards and samples were analyzed in duplicate. Insulin concentrations were calculated from the linear regression of absorbance values. Subsequently, HOMA‐IR was calculated using the following formula: HOMA‐IR = [Fasting plasma insulin (μIU/ml) × FBC (mg/dl)] /405.

### 2.6. Microcomputed Tomography (Micro‐CT)

Micro‐CT scanning was performed using a SkyScan 1272 device (Bruker, Kontich, Belgium) with the following settings: camera pixel size 7.4 μm, camera‐to‐source distance 273 mm, object‐to‐source distance 199 mm, source voltage 92 kV, source current 108 μA, and image pixel size 21 μm. Each scan lasted ~1 h and 16 min, respectively. Beam hardening correction was applied at 0.11 mm Cu as per the manufacturer guidelines.

**Figure 2:**
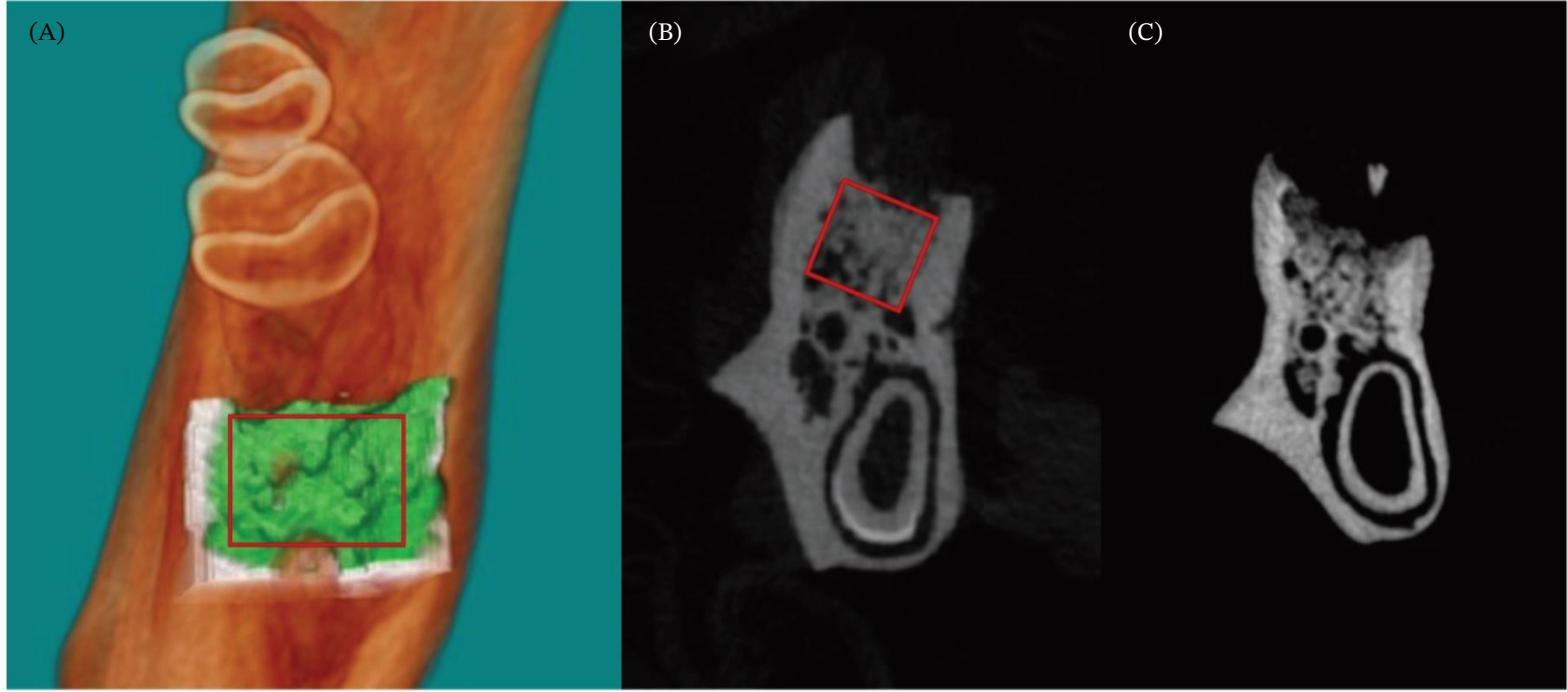
Micro‐CT analysis of extraction socket healing. (A) Three‐dimensional reconstruction showing the region of interest. (B) Frontal section image of the C28 (control) group. (C) Frontal section image of the D28 (diabetic) group. (Red box indicates ROI boundaries).

The micro‐CT data were reconstructed into 3D grayscale images by adjusting X‐ray attenuation coefficients to reflect bone architecture, following manufacturer recommendations with modifications to enhance image quality using NRecon v1.7.4.2 software (SkyScan, Leuven, Belgium). Regions of interest (ROIs) were delineated using CTAn v1.18 software (SkyScan, Leuven, Belgium), excluding the cortical bone and primary spongiosa. The ROI was defined longitudinally from the distal alveolar bone crest of M1 to a depth of 1.9 mm and horizontally as a box‐shaped area encompassing the extraction socket, measuring 2.4 mm mesiodistally and 1.2 mm buccolingually (Figure 2). The region was analyzed for volumetric parameters, including bone volume/tissue volume (%), trabecular thickness (mm), trabecular number, trabecular separation (mm), and total porosity (%).

### 2.7. Immunohistochemistry

Specimens blinded to the researcher underwent decalcification in 12.5% EDTA (pH 7.4) for 8 weeks. They were then paraffin‐embedded, sectioned at a 5 μm thickness, and washed with PBS. Endogenous peroxidase activity was blocked by incubation with 3% H2O2 for 10 min. After serum blocking, sections were incubated overnight at 4°C with mouse monoclonal antibodies: RANKL (Santa Cruz, sc‐377079, 1:100), OPG (Santa Cruz, sc‐390518, 1:100), IL‐17 (Santa Cruz, sc‐374218, 1:200), and Del‐1 (Santa Cruz, sc‐293337, 1:200). Secondary biotinylated antibodies were applied for 1 h at room temperature. Visualization was done using 3,3‐diaminobenzidine, and sections were counterstained with hematoxylin and eosin. Slides from the middle third of the alveolar socket were examined at ×100 magnification under a Primo Star light microscope (Zeiss, Oberkochen, Germany).

Three independent researchers, blinded to the sample identities, evaluated and counted the stained sections under light microscopy. A combined scoring system was used according to the established guidelines [18]. Immunopositivity in bone tissue was graded based on staining percentage: 0 = no staining; 1 = <25%; 2 = 25%–50%; 3 = 50%–75%; and 4 = >75%. Final scores were determined by consensus in cases of discordance. A weighted kappa value above 0.60 was considered to indicate substantial interobserver agreement [19].

### 2.8. Statistical Analysis

Statistical analyses were conducted using the IBM SPSS Statistics Version 24.0. Data were checked for normality and presented as the mean ± standard deviation and confidence intervals. Normally distributed data were analyzed with Student’s *t*‐test for two‐group comparisons and one‐way ANOVA with the Tukey post‐hoc test for multiple groups. OGTT glucose data were analyzed with repeated measures ANOVA and Bonferroni correction. Bivariate correlations were performed among Del‐1, IL‐17, OPG, and RANKL variables. Statistical significance was set at *p*  < 0.05.

## 3. Results

### 3.1. Systemic Parameters and Observations

Body weight gain and energy intake were initially affected by streptozotocin administration and tooth extraction but gradually increased toward the end of the experiment. Water consumption increased significantly following T2D induction, from 31.6 mL in the first week to 89.3 mL in the final week (*t*‐test, *p* < 0.001).

OGTT results revealed significant differences between the control and diabetic groups at all time points (*t*‐test, *p* < 0.001). The fasting glucose level in the control group was 77 mg/dL (95% CI: 52.3–101.6), whereas the diabetic group had a mean of 287.9 mg/dL (95% CI: 226.6–349.2). The glucose increase following glucose loading was 34.6 mg/dL (95% CI: 11.3–57.8) in the control group and 157.9 mg/dL (95% CI: 92.6–223.2) in the diabetic group (*t*‐test, *p* = 0.03).

### 3.2. ELISA and Biochemical Analysis

HOMA‐IR values were significantly higher in the diabetic group compared to the control group (*t*‐test, *p* < 0.001), with means of 4.9 (SD 1.7) and 1.18 (SD 0.3), respectively. HOMA‐IR increased from day 7 to day 14. Although no significant difference was observed between days 14 and 28, both timepoints showed higher values than day 7 (Tukey, *p* = 0.018 and *p* < 0.001, respectively).

### 3.3. Micro‐CT

On day 28, micro‐CT analysis showed greater bone formation in the control subgroup (C28), as indicated by a significantly higher bone volume/tissue volume (BV/TV) ratio: 67.41% (SD 2.69) vs. 55.95% (SD 6.61) in the diabetic subgroup (D28) (*t*‐test, *p* = 0.007). Trabecular separation (Tb.Sp) was significantly wider in D28 (0.42 mm) compared to C28 (0.32 mm) (*t*‐test, *p* = 0.036). Total porosity (Po(tot)) was also higher in D28 (44.03%) than C28 (27.8%) (*t*‐test, *p* = 0.005). No significant differences were found in trabecular thickness (Tb.Th) or number (Tb.N) between groups (*t*‐test, *p* = 0.630 and *p* = 0.632, respectively) (Table 2).

**Table 2:** Microcomputed tomography results on day 28.

Parameters	C28 (*n* = 5)	D28 (*n* = 5)	Difference	*P* value
	Mean (SD)	Mean (SD)	95% CI	
BV/TV (%)	67.41 (2.69)	55.95 (6.61)	(4.09, 18.83)	***0.007***
Tb.Th (mm)	0.19 (0.10)	0.17 (0.07)	(−0.10, 0.15)	0.630
Tb.N	2.04 (0.18)	2.09 (0.15)	(−0.30, 0.19)	0.632
Tb.Sp (mm)	0.32 (0.03)	0.42 (0.08)	(−0.19, −0,00)	***0.036***
Po(tot) (%)	27.8 (6.81)	44.03 (6.61)	(−25.9, −6.34)	***0.005***

*Note:* Parameters were evaluated with micro‐CT for control and diabetic subgroups of 28 days of alveolar socket healing. Bone volume/tissue volume (BV/TV), trabecular thickness (Tb.Th), trabecular number (Tb.N), trabecular separation (Tb.Sp), and total porosity (Po[tot]). 95% confidence intervals of the difference are shown. Bold is used to highlight the Student’s *t*‐test *p* value below 0.05.

### 3.4. Immunohistochemical Findings

**Figure 3:**
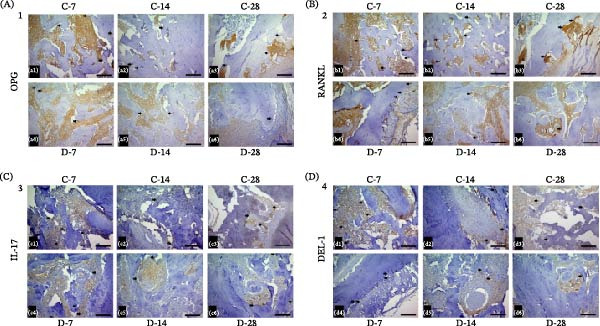
Light microscopy images of the alveolar socket healing for subgroups C7, C14, C28, D7, D14, and D28 on days 7, 14, and 28 in means of OPG (A), RANKL (B), IL‐17 (C), and Del‐1 (D) staining, respectively (bar = 100 µm). Arrows indicate staining.

**Figure 4:**
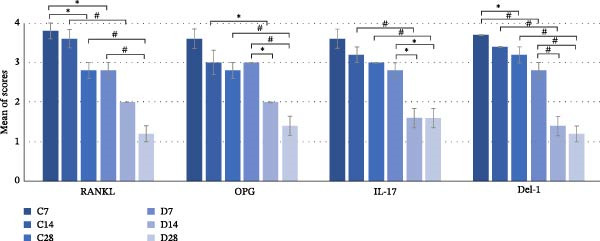
Mean immunopositivity scores of the alveolar sockets for subgroups C7, C14, C28, D7, D17, and D28, respectively, on days 7, 14, and 28 in terms of OPG, RANKL, IL‐17, and Del‐1. Asterisks indicate a *p* value is less than 0.05, and a hash sign indicates a *p* value is less than 0.001.

As shown in Figure 3, RANKL and OPG were observed in bone cells (osteoblasts and osteocytes), Del‐1 was detected in vessel walls within the bone marrow, and IL‐17 was present in both the bone tissue and bone marrow. Protein expression was consistently higher in control subgroups than in diabetic subgroups at matched time points (Tukey, *p* < 0.05 for all) (Figure 4). In the control group, staining intensity gradually decreased from day 7 to day 28 but remained visible in vascular regions for IL‐17 and Del‐1 and on osteoblasts for RANKL and OPG. At day 28, Del‐1 staining was still detectable in the newly formed bone of the C28 subgroup. In contrast, immunoreactivity in the diabetic group was markedly reduced across all markers (Figure 4).

### 3.5. Protein Correlations

RANKL/OPG means ratios did not differ among subgroups, but the main control group showed a higher ratio (1.1) compared to the main diabetic group (0.95) (*t*‐test, *p* = 0.022).

There was a positive correlation between OPG and RANKL (*r* = 0.85, *n* = 30, *p*  < 0.001), and between Del‐1 and IL‐17 (*r* = 0.678, *n* = 30, *p*  < 0.001). Investigated protein levels were generally positively correlated, as in reduced in the T2D subgroups and induced in the control subgroups, collectively (*p*  < 0.001).

## 4. Discussion

Impaired wound healing is a well‐established consequence of diabetes and is known to prolong the tissue regeneration process [20]. This also extends to postextraction socket healing in individuals with T2D [21]. However, the precise mechanisms by which diabetes alters bone healing remain to be fully elucidated.

In the present study, we employed a widely accepted T2D rat model that mirrors human metabolic characteristics by combining an HFD to induce insulin resistance with low‐dose STZ to partially impair β‐cell function. This model does not produce insulin‐dependent diabetes but reflects hyperglycemia [16], aligning with our findings. As in previous studies using similar protocols [22, 23], our HOMA‐IR values confirmed insulin resistance.

In healthy rats, alveolar socket healing typically completes by day 28 following extraction [12]. Our micro CT findings at day 28 were comparable to previous control group results under normal conditions [12]. In line with earlier T2D research [21, 24], our diabetic group demonstrated decreased bone volume and increased porosity, echoing clinical observations such as wider socket defects and more demineralized tissue in diabetic patients and pig models.

Although both OPG and RANKL are known to be expressed by osteocytes, osteoblasts, and other stromal cells in the bone marrow [25], our immunohistochemical analysis revealed staining only in osteoblasts and osteocytes, not in the other stromal cells. This might be attributed to the technical limitations of the staining protocol or the possibility that other stromal cell expression levels were too low to be immunohistochemically detectable in our experimental model.

Overall, both OPG and RANKL expression levels were reduced in the diabetic subgroups at most time points, and the RANKL/OPG ratio was consistently higher in the control subgroups. This ratio is considered a hallmark of ongoing resorptive activity [5, 6] and may reflect a more dynamic bone healing response under normoglycemic conditions. These findings align with previous studies in Wistar rats, where diabetes was associated with suppressed osteoblastic RANKL production and a consequent decrease in the RANKL/OPG ratio [26].

Unlike Hassumi et al. [12], who reported no significant temporal changes in OPG or RANKL expression over 28 days, we observed a marked decline in RANKL levels between days 7 and 28 in control subgroups, while OPG levels remained relatively stable. This temporal change further supports the hypothesis that RANKL expression may be more dynamically regulated during early healing under healthy conditions.

Despite an extensive literature review, few studies have investigated these markers specifically within the context of alveolar socket healing under diabetic conditions, limiting our ability to draw direct comparisons. Nonetheless, our findings point toward suppressed bone turnover signaling in diabetic animals, possibly contributing to delayed or impaired healing in T2D conditions.

A recent review on IL‐17’s role in tissue repair highlights the controversy surrounding its impact on bone wound healing, with some studies identifying IL‐17 as a negative regulator and others reporting adverse outcomes following anti‐IL‐17 treatments [27]. IL‐17 appears to exert dual effects depending on the healing stage: it promotes osteoblastic differentiation in the early phases [28], while in later stages, it may contribute to bone loss by inducing pro‐osteoclastogenic cytokines such as TNF‐α and RANKL from osteoblasts [29]. In our study, IL‐17 expression was significantly higher in the control group compared to the diabetic group on postoperative day 7, which may support the hypothesis that IL‐17 plays a beneficial role in the early stages of bone healing under normoglycemic conditions. Although IL‐17 immunopositivity declined over time, it remained consistently higher in the control groups relative to those in the diabetic groups. However, the lack of studies investigating IL‐17 expression during alveolar socket healing under diabetic conditions limits direct comparison with our results.

In this study, Del‑1 expression was confined to vascular regions within the bone marrow, and this restriction aligns with previous findings showing that Del‑1 is predominantly secreted by endothelial cells and primarily localized in vascular compartments [30, 31].

Del‑1 has also been shown to support both angiogenesis and osteogenic differentiation, indicating its dual role in coordinating vascular and bone tissue responses crucial for effective alveolar bone healing [32, 33]. Accordingly, the reduced staining observed in diabetic groups compared to controls may point to a potential link between lower Del‑1 expression and insufficient bone healing in T2D, as reflected by the micro‐CT findings. However, this potential association requires further investigation through studies focusing on the underlying mechanisms involved in diabetic bone healing.

There is a well‐established inverse relationship between Del‑1 and IL‑17 in the literature, with IL‑17 known to downregulate Del‑1 expression in inflammatory bone loss models and diabetic periodontitis patients [11, 34]. Conversely, in our study of alveolar socket healing, we observed a positive correlation between these proteins. This suggests that the regulatory dynamics between Del‑1 and IL‑17 may differ in the context of bone healing, potentially representing a distinctive feature of alveolar socket regeneration.

While this study sheds light on the altered protein expression during alveolar socket healing in the context of T2D, the findings require further validation. Several limitations should be acknowledged. First, as immunohistochemistry is a semiquantitative technique without digital image analysis, future studies should incorporate quantitative methods at the tissue level, as well as gene expression analysis. Second, the relatively small sample size may limit the statistical power for detecting subtle differences. Third, micro‐CT evaluation was performed only at day 28, precluding the longitudinal assessment of structural changes. Fourth, only male animals were used, and given the known effects of estrogen on bone metabolism, the results may not be directly generalizable to females. Finally, while the HFD/STZ rat model closely mimics human T2D metabolic characteristics, inherent differences in bone healing kinetics, metabolic rates, and lifespan between rats and humans limit direct clinical translation. Human alveolar socket healing occurs over 3–6 months compared to 28 days in rats, and the molecular dynamics may differ accordingly. Nevertheless, this animal model provides valuable mechanistic insights that can inform hypothesis generation for future clinical studies.

## 5. Conclusion

This study demonstrates that T2D significantly alters the expression of key regulatory proteins involved in alveolar socket healing, with potential implications for clinical practice. The observed suppression of bone turnover markers suggests that diabetic patients may require extended healing periods before implant placement. Furthermore, the identified proteins—particularly Del‐1 and the RANKL/OPG axis—represent potential therapeutic targets for enhancing bone healing in diabetic individuals. Future research should explore whether local delivery of Del‐1 or modulation of the RANKL/OPG pathway could improve the socket healing outcomes in this vulnerable population.

**Nomenclature**
Del‐1: Developmental endothelial locus‐1
IL‐17: Interleukin 17
micro‐CT: Microcomputed tomography
OPG: Osteoprotegerin
RANKL: Activator of the nuclear factor kappa beta ligand
T2D: Type 2 diabetes
OGTT: Oral glucose tolerance test
HOMA‐IR: Homeostatis model assessment of insulin resistance.

## Author Contributions

Kübra Güler, Emine Pirim Görgün, Özhan Karataş, and Serkan Bolat participated in designing the study. Kübra Güler, Özhan Karataş, and Serkan Bolat participated in generating the data for the study, participated in gathering the data for the study, and participated in the analysis of the data. Kübra Güler wrote the majority of the original draft of the paper and had access to all the raw data of the study. Özhan Karataş and Serkan Bolat participated in writing the paper. Kübra Güler and Emine Pirim Görgün reviewed the pertinent raw data on which the results and conclusions of this study were based.

## Funding

This work was supported by the Sivas Cumhuriyet University Scientific Research Projects Coordination Unit (Grant DİŞ‐2021‐270).

## Disclosure

All authors read and approved the final manuscript.

## Ethics Statement

This manuscript received ethical approval from the Sivas Cumhuriyet University Animal Experiments Ethics Committee (Approval Number: 65202830‐050.04.04‐484). Consent to participate is not applicable.

## Consent

The authors have nothing to report.

## Conflicts of Interest

The authors declare no conflicts of interest.

## Data Availability

The datasets used and/or analyzed during the current study are available from the corresponding author upon reasonable request.
